# Vitamin D Deficiency Is Associated with Muscle Atrophy and Reduced Mitochondrial Function in Patients with Chronic Low Back Pain

**DOI:** 10.1155/2019/6835341

**Published:** 2019-06-02

**Authors:** Katarzyna Patrycja Dzik, Wojciech Skrobot, Katarzyna Barbara Kaczor, Damian Jozef Flis, Mateusz Jakub Karnia, Witold Libionka, Jedrzej Antosiewicz, Wojciech Kloc, Jan Jacek Kaczor

**Affiliations:** ^1^Department of Neurobiology of Muscle, Gdansk University of Physical Education and Sport, K. Gorskiego 1, 80-336 Gdansk, Poland; ^2^Department of Kinesiology, Gdansk University of Physical Education and Sport, K. Gorskiego 1, 80-336 Gdansk, Poland; ^3^Department of Bioenergetics and Physiology of Exercise, Medical University of Gdansk, 80-211 Gdansk, Poland; ^4^Department of Bioenergetics and Nutrition, Gdansk University of Physical Education and Sport, K. Gorskiego 1, 80-336 Gdansk, Poland; ^5^Department of Neurosurgery, Copernicus Hospital, Gdansk, Poland; ^6^Department of Biochemistry, Gdansk University of Physical Education and Sport, K. Gorskiego 1, 80-336 Gdansk, Poland; ^7^Department of Neurology and Neurosurgery, University of Warmia and Mazury in Olsztyn, Poland

## Abstract

Recent studies show that vitamin D deficiency may be responsible for muscle atrophy. The purpose of this study was to investigate markers of muscle atrophy, signalling proteins, and mitochondrial capacity in patients with chronic low back pain with a focus on gender and serum vitamin D level. The study involved patients with chronic low back pain (LBP) qualified for posterior lumbar interbody fusion (PLIF). Patients were divided into three groups: supplemented (SUPL) with vitamin D (3200 IU/day for 5 weeks), placebo with normal levels of vitamin D (SUF), and the placebo group with vitamin D deficiency (DEF). The marker of muscle atrophy including atrogin-1 and protein content for IGF-1, Akt, FOXO3a, PGC-1*α*, and citrate synthase (CS) activity were determined in collected multifidus muscle. In the paraspinal muscle, IGF-1 levels were higher in the SUF group as compared to both the SUPL and DEF groups (*p* < 0.05). In the SUPL group, we found significantly increased protein content for pAkt (*p* < 0.05) and decreased level of FOXO3a (*p* < 0.05). Atrogin-1 content was significantly different between men and women (*p* < 0.05). The protein content of PGC-1*α* was significantly higher in the SUF group as compared to the DEF group (*p* < 0.05). CS activity in the paraspinal muscle was higher in the SUPL group than in the DEF group (*p* < 0.05). Our results suggest that vitamin D deficiency is associated with elevated oxidative stress, muscle atrophy, and reduced mitochondrial function in the multifidus muscle. Therefore, vitamin D-deficient LBP patients might have reduced possibilities on early and effective rehabilitation after PLIF surgery.

## 1. Introduction

Skeletal muscle atrophy occurs when the normal balance between synthesis and degradation of muscle structural proteins is disturbed. Chronic low back pain (LBP), one of the most prevalent musculoskeletal disorders in modern society [1], leads to the atrophy of paraspinal muscles [2]. Muscle atrophy Fbox (MAFbx/atrogin-1), was identified as a gene of muscle specific ubiquitin ligase (E3). This ligase, along with muscle RING finger 1 (MuRF1), is responsible for the degradation of the muscle structural proteins in atrophied skeletal muscles that are caused by immobilization [3], disuse, dietary restriction, aging, cancer, etc. [4–6]. In particular, these genes have been known to be significantly responsible for muscle atrophy since their inhibition reduces muscle atrophy caused by denervation. Additionally, they have been shown to play a key role in the induction of muscle atrophy in multiple animal disuse models [4, 5, 7]. Notwithstanding this data, the exact mechanism underlying muscle atrophy has not been fully elucidated.

LBP may be caused by different factors including the loss of lumbar spinal stability through nonsufficient activation of the deep lumbar stabilizing muscles such as the multifidus muscle [8]. Hence, reduced activation of the multifidus muscle is a major cause of its progressive muscle atrophy and upregulation of atrogin-1 gene expression. The serine/threonine-specific protein kinase (Akt)/forkhead box O3 (FOXO3) axis controls the expression of atrogin-1 gene [9]. FOXO transcription factors are thought to control half of the genes identified in the molecular “common atrophy blueprint” present in different atrophy types [10, 11]. Akt is a protein kinase, which is important in signalling pathways involved in protein synthesis and skeletal muscle growth [12]. Also, overproduction of reactive oxygen species (ROS), disturbed redox status, and a weakened antioxidant defense system are known as the major contributing factors toward atrophy [13]. Recently, we demonstrated that vitamin D deficiency is associated with higher oxidative stress and elevated activity of antioxidant enzymes in the paraspinal muscle of patients with LBP [14].

Vitamin D seems to act as a multifunctional regulator in skeletal muscle [15]. Vitamin D contributes to maintain musculoskeletal health in healthy subjects as well as in patients who display the combination of paraspinal muscle wasting and weakness such as LBP patients [16]. Cross-sectional studies found a positive association between vitamin D status and total or appendicular muscle mass in men and women [17–19]. The actions of the vitamin D hormone are mediated by the vitamin D receptor (VDR), a ligand-activated transcription factor that controls gene expression [20, 21]. An increasing number of studies in both nonhuman and human skeletal muscle cells report that the actions of vitamin D are also mediated by the VDR located within skeletal muscle cells [22–24]. Interestingly, the recent study shows that pharmacologically induced muscle loss in VDR^−/−^ mice is greater in slow muscles, such as the multifidus muscle, than in fast muscles [25]. The exact mechanism of action of vitamin D in the muscle remains unknown. Insulin-like growth factor 1 (IGF-1), an anabolic hormone, has been shown to positively correlate with 25-hydroxy vitamin D serum level [26]. Therefore, we assume that vitamin D deficiency might be associated with downregulated IGF-1 in the atrophied skeletal muscle. Recently, we have reported that long term of vitamin D deficiency leads to VDR ablation, oxidative stress, and consequence mitochondrial dysfunction, which induces muscle atrophy [27].

The purpose of this study was to estimate and compare the levels of selected markers of muscle atrophy, signalling proteins, and mitochondrial capacity in the skeletal muscles of patients deficient in and with normal vitamin D level, and patients supplemented with vitamin D or placebo. Moreover, based on the recent data [14], we assumed that the possible mechanism of vitamin D in the prevention of muscle atrophy may be mediated through oxidative stress and the IGF-1/Akt/FOXO3 pathway. Specifically, we propose that muscle atrophy linked with serum vitamin D deficiency is associated with a reduction of IGF-1 and deactivation and activation of Akt and FOXO3. Furthermore, normalized levels of serum vitamin D would ameliorate relative muscle atrophy and maintain physiological mitochondrial function.

## 2. Materials and Methods

### 2.1. LBP Patients

The study population was previously described by Dzik and coworkers [14]. Briefly, nineteen women and nineteen men participated in the study. All patients were Caucasian. Pregnant or lactating women were not included. All patients had experienced chronic LBP secondary to the degenerative disease and general instability and were qualified for lumbar spine surgery utilizing static or dynamic implants (posterior lumbar interbody fusion (PLIF)). There were no significant differences in pain duration and intensity between genders. In all cases, the LBP causes were nonspecific and mechanical. All subjects gave their informed consent for inclusion before they participated in the study. The study was conducted in accordance with the Declaration of Helsinki, and the protocol was approved by the local institutional Bioethical Committee in Gdansk (No. NKBBN/120/2012).

### 2.2. Study Design

The study design was previously described by Dzik and coworkers [14]. Briefly, patients were randomly assigned to the group supplemented with 3200 IU of 25(OH)D_3_/day for 5 weeks (SUPL, *n* = 14) or the placebo group supplemented with vegetable oil. Blood samples were taken at baseline and after 5 weeks of supplementation for the determination of serum vitamin D concentration. Based on serum vitamin D concentration, patients from the placebo group were divided into two groups: the placebo group with normal concentration of vitamin D (SUF, *n* = 10) with 25(OH)D_3_ level above 50 nmol/L and the placebo group with vitamin D deficiency (DEF, *n* = 14) with 25(OH)D_3_ serum level between 30 and 49 nmol/L [28]. After 5 weeks of supplementation, multifidus muscle samples were obtained from all the patients during PLIF surgery. Patients' characteristics are summarized in Table 1.

### 2.3. Blood Analysis and Collection

Blood samples were taken at baseline and after 5 weeks of supplementation. The samples were centrifuged at 2000 *g* for 10 min at 4°C. The separated serum samples were frozen and kept at -80°C until later analysis. The tubes containing the serum samples were number-coded in order to blind the laboratory personnel regarding the treatment group and the sequence of sample collection. IGF-1 in serum was measured with an immunoassay kit (DG100, R&D Systems, USA) according to the manufacturer's instructions.

### 2.4. Human Muscle Sample

After 5 weeks of supplementation, multifidus muscle samples were obtained from all patients during PLIF surgery. All muscle samples were taken between the tenth thoracic and fifth lumbar vertebrae. 40-150 mg multifidus muscle specimens were collected and immediately frozen at -80°C.

### 2.5. Muscle Homogenization

The tissue samples were reconstituted in ice-cold lysis buffer containing 50 mM Tris-HCl, 1 mM EDTA, 1.15% KCl, 0.5 mM DTT, 0.2% protease inhibitor cocktail (Sigma-Aldrich, P834), and phosphatase inhibitor tablets PhosSTOP (Roche, Italy). The final homogenate concentration was 8%. The samples were centrifuged at 750 *g* for 10 min at 4°C, and the supernatant was divided into serial aliquots for enzyme activity, enzyme-linked immunosorbent assay (ELISA), and western blot (WB) measurements. Samples for WB were centrifuged at 16000 *g* and for ELISA at 5000 *g*. Protein concentration was determined using the Bradford protein assay (Sigma-Aldrich, B6916) according to the manufacturer's instructions.

### 2.6. Assays: Muscle Analysis

Insulin-like growth factor 1 (IGF-1) and atrogin-1 in muscle homogenates were determined using immunoassay kits (IGF-1-SEA050Hu. Cloud Clone Corporation; atrogin-1- EH4228, Fine Test), according to the manufacturer's instructions.

### 2.7. Mitochondrial Citrate Synthase Activity

Citrate synthase (CS) activity was measured at 37°C according to De Lisio et al. [29]. Briefly, 30 *μ*l of supernatant (diluted to 4% final concentration; 750 *g*) was added to 850 *μ*l of buffer (0.1 M Tris-HCl, 5 mM EDTA, 0.05% Triton-X100, pH 8.1), plus 100 *μ*l of freshly made DTNB (1 mM), 10 *μ*l acetyl-CoA (10 *μ*M), and 10 *μ*l of freshly made oxaloacetic acid (10 mM) to initiate the reaction. The reactions were conducted in duplicate, and absorbance was read at 412 nm in a spectrophotometer (CE9200, Cecil Instruments Limited, Cambridge, UK). CS activity was expressed as nmol/min/mg of protein.

### 2.8. Western Blotting

Equal amounts of total tissue lysates were separated on either 4-20%, 30 *μ*l Mini-PROTEAN TGX™ gels (Bio-Rad Laboratories, USA) or 10% SDS-polyacrylamide gel electrophoresis (SDS-PAGE) and transferred onto a polyvinylidene difluoride (PVDF) membrane. The membranes were then blocked with a solution containing 10 mM Tris-buffered saline, 0.05% Tween 20, and 5% nonfat dry milk or 5% bovine serum albumin (BSA) (Sigma-Aldrich) and then incubated with primary antibodies including PGC-1*α* (Santa Cruz, sc-13067, dilution 1 : 500), Akt 1/2/3, (Santa Cruz, sc-8312, dilution 1 : 500), P-Akt 1/2/3 (Ser^473^) (Santa Cruz, sc-7985-R, dilution 1 : 500), FoxO3a (Cell Signaling, 2497, dilution 1 : 500), P-FoxO3a (Abcam, ab154786, dilution 1 : 500), Fbx32 (Abcam, ab168372, dilution 1 : 1000), and *β*-tubulin (Cell Signaling, 2146, dilution 1 : 500) over night at 4°C. The membranes were treated with secondary anti-rabbit and anti-mouse antibody (dilution 1 : 20000) for 1 h at room temperature. Following treatment with the appropriate secondary antibody, the bands were visualized using ImageQuant LAS 500 (GE Healthcare). The changes in the protein level were quantified by a densitometric method using the LASImage software. *β*-Tubulin was used as a lane loading control. The immunoblotting was performed at least two times.

### 2.9. Statistical Analysis

Statistical analyses were performed using a software package (Statistica v. 13.1, StatSoft Inc., Tulsa, OK, USA). The results are expressed as the mean ± SEM. The differences between men and women in the same group were tested by Student's *t*-test. To identify significant differences between groups, results were analyzed by ANOVA followed by the Least Significant Difference (LSD) test. Differences with a *p* value of at least *p* ≤ 0.05 were considered statistically significant.

## 3. Results

Data with patients' serum 25(OH)D_3_ level before and after the supplementation were previously published [14] and are summarized in Table 1. Briefly, serum 25(OH)D_3_ level was significantly different between the placebo groups, the DEF and SUF groups, both before and after the supplementation period. Five weeks of supplementation with a daily dose of 3200 IU vitamin D_3_ raised serum vitamin D level by an average of 53 nmol/L in the SUPL group and placed the level of serum 25(OH)D_3_ above 87 nmol/L, which is 12 nmol/L higher than the level indicated as a threshold for optimal vitamin D level for adults [28, 30].

**Figure 1:**
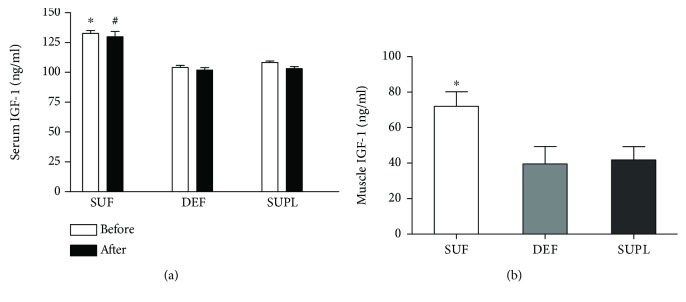
The level of IGF-1 in (a) serum and (b) skeletal muscle of LBP patients. Results were expressed as the mean ± SEM. (a) DEF (*n* = 13), SUF (*n* = 9), and SUPL (*n* = 14). (b) SUF (*n* = 4), DEF (*n* = 6), and SUPL (*n* = 8). ^∗^ *p* < 0.05—difference between the indicated result/mean and DEF and SUPL groups at the same time point. ^#^ *p* < 0.005—difference between the indicated result/mean and DEF and SUPL groups at the same time point.

Circulating IGF-1 content was significantly higher in the SUF group as compared to both the DEF and SUPL groups before and after the supplementation. Before the supplementation, serum IGF-1 content was 108.3 ± 4.2, 104.1 ± 6.3, and 132.6 ± 7.4 ng/mL in the SUPL, DEF, and SUF groups, respectively (Figure 1(a), *p* < 0.05). After the supplementation, serum IGF-1 level was as 103.1 ± 6.5 ng/mL in the SUPL group, 101.9 ± 7.3 ng/mL in the DEF group, and 129.7 ± 13.3 ng/mL in the SUF group (Figure 1(a), *p* < 0.05). We did not observe any difference neither before and after the supplementation nor between men and women in particular groups. Muscle IGF-1 concentration was the highest in the SUF group, 71.9 ± 8.2 ng/mL. It was significantly lower in the DEF and SUPL groups (Figure 1(b), *p* < 0.05). The level of IGF-1 in the DEF group was 39.5 ± 9.8 and 41.8 ± 7.5 ng/mL in the SUPL group. We did not find any difference between men and women in muscle IGF-1 levels.

**Figure 2:**
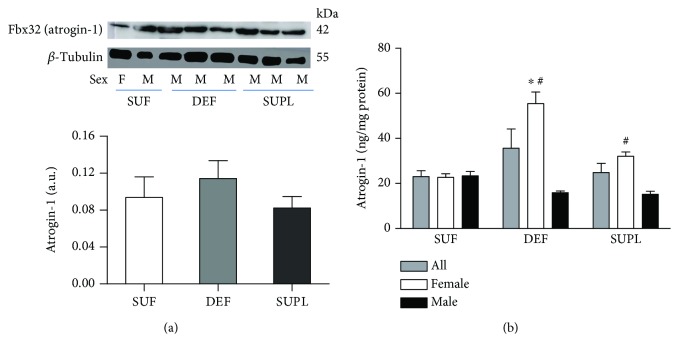
The level of atrogin-1 (Fbx32) visualized by representative western blotting (a) and measured with ELISA (b) in all LBP patients, female and male in skeletal muscle. Changes in WB protein densitometry levels were normalized against *β*-tubulin. a.u.: arbitrary units; F: female; M: male. Results were expressed as the mean ± SEM. (a) SUF (*n* = 6), DEF (*n* = 7), and SUPL (*n* = 6). (b) SUF (*n* = 10), DEF (*n* = 12), SUPL (*n* = 14), SUF F (*n* = 5), SUF M (*n* = 5), DEF F (*n* = 6), DEF M (*n* = 6), SUPL F (*n* = 8), and SUPL M (*n* = 6). ^∗^ *p* < 0.05—difference between the indicated result/mean and female SUF group. ^#^ *p* < 0.05—difference between the indicated result/mean and men in the same group.

Western blotting analysis of the muscle atrophy marker Fbx32 (atrogin-1) showed that in the DEF atrogin-1 content was 38.7% higher than in the SUP group and 22% higher than in the SUF group (Figure 2(a)). The muscular concentration of atrogin-1, measured with ELISA, was the highest in the DEF group (35.7 ± 8.5 ng/mg protein). In the SUF and SUPL groups, the content of atrogin-1 was 23.1 ± 2.6 and 24.8 ± 4.1 ng/mg, respectively (Figure 2(b)). There was a significant difference in atrogin-1 muscle content between men and women overall. Muscle atrogin-1 level was 50% higher in women as compared to men (36.9 ± 5.4 ng/mg and 17.9 ± 1.9 ng/mg, respectively). There was no difference among men in atrogin-1 content in the muscle (15.9 ± 1.8, 23.4 ± 4.2, and 15.2 ± 3.4 ng/mg protein in the DEF, SUF, and SUPL groups, respectively). However, there was a difference observed among women between the three groups. Women in the DEF group had significantly higher atrogin-1 level as compared to those in the SUF group (Figure 2(b), *p* < 0.05). Vitamin D-deficient women had an average of 55.4 ± 12.7 ng/mg, and women sufficient in vitamin D had an average of 22.7 ± 3.6 ng/mg. Among women supplemented with vitamin D, the protein content of atrogin-1 was 32.0 ± 5.6 ng/mg. Furthermore, women in the DEF and SUPL groups had significantly higher atrogin-1 content as compared to the corresponding groups of men (Figure 2(b), *p* < 0.05). There was no difference between men and women in the SUF group.

**Figure 3:**
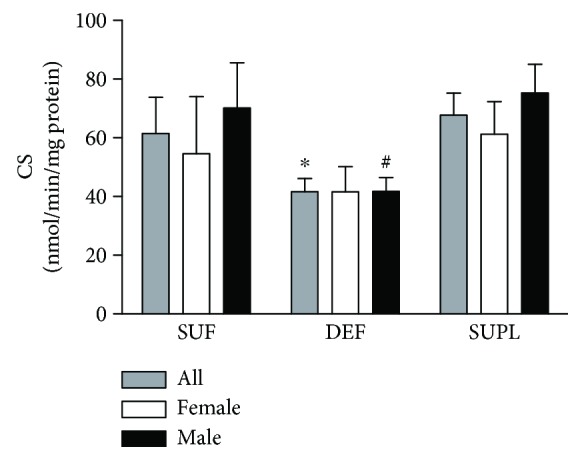
The CS enzyme activity in the skeletal muscle of all LBP patients. Results were expressed as the mean ± SEM. SUF (*n* = 9), DEF (*n* = 13), SUPL (*n* = 13), SUF F (*n* = 5), SUF M (*n* = 4), DEF F (*n* = 6), DEF M (*n* = 7), SUPL F (*n* = 7), and SUPL M (*n* = 6). ^∗^ *p* < 0.05—difference between the indicated result/mean and SUPL in all patients. ^#^ *p* < 0.05—difference between the indicated result/mean and men in the SUPL group.

The activity of citrate synthase (CS) in the muscle, which is commonly used as a marker of mitochondrial function [31], was significantly higher in the SUPL group when compared with the DEF group. In the SUF group, CS activity tended to be higher than in the DEF group, but the difference was not significant. The activity of CS in all patients was 67.7 ± 7.4, 61.5 ± 12.3, and 41.6 ± 4.5 nmol/min/mg of protein in the SUPL, SUF, and DEF groups, respectively (Figure 3, *p* < 0.05). Among women, we did not observe any differences between the groups, whereas in men there was significantly higher CS activity in the SUPL group when compared with the DEF group (Figure 3, *p* < 0.05).

**Figure 4:**
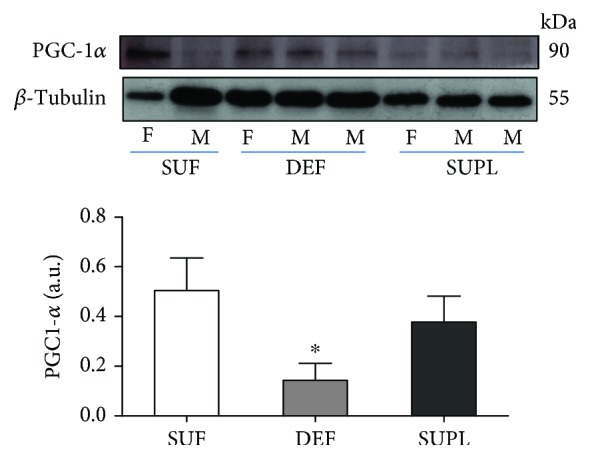
The content of PGC1-*α* in the muscles of LBP patients, visualized by representative western blotting. Changes in all presented protein densitometry levels were normalized against *β*-tubulin. a.u.: arbitrary units; F: female; M: male. Results were expressed as the mean ± SEM. SUF (*n* = 5), DEF (*n* = 4), and SUPL (*n* = 6). ^∗^ *p* < 0.05—difference between the indicated result/mean and SUF.

The protein content of the mitochondrial biogenesis transcription factor—PGC-1*α*—was significantly higher in the SUF group as compared to the DEF group (Figure 4, *p* < 0.05). In the SUPL group, the PGC-1*α* content was also higher than in the DEF group, but the difference did not reach the significance.

**Figure 5:**
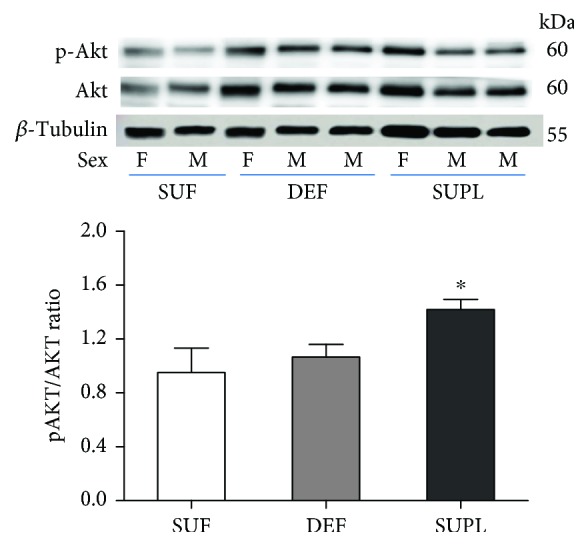
The ratio of pAkt/Akt content in the muscles of LBP patients, visualized by representative western blotting. Changes in all presented protein densitometry levels were normalized against *β*-tubulin. F: female; M: male. Results were expressed as the mean ± SEM. SUF (*n* = 4), DEF (*n* = 6), and SUPL (*n* = 6). ^∗^ *p* < 0.05—difference between the indicated result/mean and other groups.

**Figure 6:**
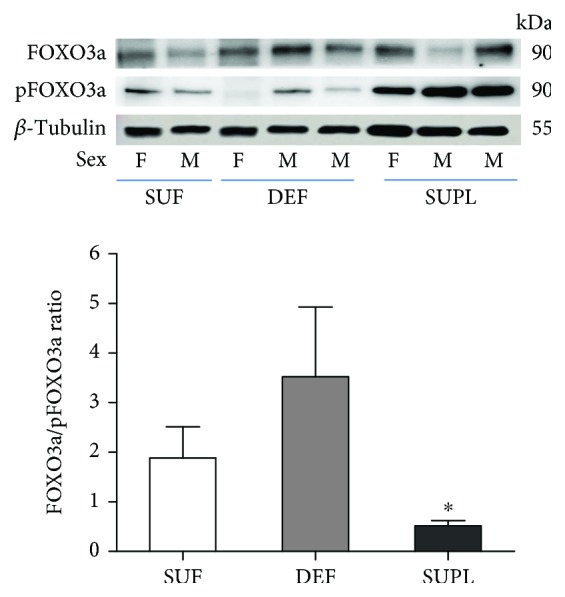
The ratio of FOXO3a/pFOXO3a content in the muscles of LBP patients, visualized by representative western blotting. Changes in all presented protein densitometry levels were normalized against *β*-tubulin. F: female; M: male. Results were expressed as the mean ± SEM. SUF (*n* = 4), DEF (*n* = 6), and SUPL (*n* = 6). ^∗^ *p* < 0.05—difference between the indicated result/mean and DEF.

To determine the possible mechanism of vitamin D on muscle atrophy, we investigated the phosphorylation states of Akt and FOXO3a. The protein content of phosphorylated Akt (pAkt) and phosphorylated FOXO3a (pFOXO3a) was similar in the DEF and SUF groups. In the SUPL group, we observed significantly higher levels of pAkt (Figure 5, *p* < 0.05) and decreased level of FOXO3a (Figure 6, *p* < 0.05).

## 4. Discussion

The main findings of our study are that LBP patients with serum vitamin D deficiency show attenuated CS activity, increased content of atrogin-1, and decreased PGC-1*α* protein content in the multifidus muscle. In addition, we noticed higher IGF-1 content, in both serum and muscle, in patients with sufficient vitamin D level. Moreover, we observed significantly increased level of pAkt and decreased level of FOXO3a in patients supplemented with vitamin D. Together, our results suggest that the action of vitamin D in the muscle may be triggered through either the Akt/FOXO3a pathway or PGC-1*α* as a result of ROS generation.

Hitherto, the interplay between vitamin D and IGF-1, a hormone which displays an anabolic effect on skeletal muscle, has been well described in reference to their circulation level. Wei and coworkers showed that IGF-1 caused an increase in the blood levels of 1,25(OH)_2_D_3_, the hormonally active vitamin D metabolite, by stimulating the expression and activity of the hydroxylase-1*α* that produces 1,25(OH)_2_D_3_ in the kidney [32]. Moreover, when vitamin D was administered to humans, IGF-1 levels in the blood increased [33]. On the other hand, another study showed that one year of high-dose vitamin D supplementation did not significantly alter serum IGF-1 among women at a high risk of breast cancer [34] nor in prediabetes subjects [35]. Our results revealed that patients in the SUF group had higher serum IGF-1 level than patients in the DEF and SUPL groups, both before and after the supplementation period. Patients with normal vitamin D levels presented with approximately a 20% higher IGF-1 serum concentration than those deficient in vitamin D or those supplemented with it. However, we did not observe any changes regarding supplementation itself within particular groups. Muscle IGF-1 content was higher in patients with sufficient serum vitamin D level as compared to the other groups, and this was consistent with circulating IGF-1 level. Surprisingly, we did not find any difference between patients supplemented with vitamin D and patients deficient in it. Recently, Hayakawa and coworkers showed that IGF-1 is not directly affected by 1,25(OH)_2_D_3_ in the skeletal muscle. They suggested that vitamin D stimulated IGF-1 production in tissues other than the skeletal muscle and that the induced IGF-1 could enter systemic circulation and exert hypertrophic effects on the muscle tissue or supportive effects on muscle function [15]. In this study, we showed elevated serum and muscle IGF-1 content in LBP patients sufficient in vitamin D. The lack of an increase in IGF-1 in the SUPL group suggests that either IGF-1 is not directly influenced by vitamin D or its induction is time dependent. This raises an interesting point that should be addressed in future studies. Notably, how long sufficient vitamin D level must be present in circulation in order to increase IGF-1 muscle content in humans who were previously deficient in vitamin D? It is important to note that we do not find any correlation between IGF-1 and atrogin-1, which suggests that the mechanism of action of vitamin D on skeletal muscle atrophy might involve other factors.

In the present study, we analyzed the muscle content of atrogin-1 and showed that atrogin-1 was the highest in patients deficient in vitamin D and lowest in patients sufficient in it. Vitamin D supplementation seems to repel atrophic changes since we observed almost as low a content of atrogin-1 in the SUPL group as in the SUF group. Notwithstanding, these results are not significant when we consider men and women together. The present study shows that women and men respond differently to vitamin D deficiency and supplementation. Men seem to be less responsive to vitamin D in regard to paraspinal muscle atrophy. Notably, among women, there was an elevated level of atrogin-1 in the group deficient in vitamin D as compared to those sufficient in it. It seems that vitamin D deficiency escalates muscle atrophy among women. Our findings are consistent with the latest study on the effect of long-term vitamin D supplementation on the global transcriptomic profile which showed that vitamin D regulates 3.2-fold more genes in women than in men [36]. Hereby, we could detect a stronger effect of vitamin D supplementation on gene expression in women when compared to men. Moreover, we observed almost the same level of atrogin-1 among women sufficient in vitamin D as in women supplemented with vitamin D who were deficient in vitamin D at baseline and whose serum vitamin D level increased to normal levels. This observation may suggest a need for vitamin D supplementation for women in order to delay the onset muscle atrophy.

Mitochondrial dysfunction in vitamin D-deficient individuals was attributed to intramitochondrial calcium deficiency [37] or deficient enzyme function of the oxidative pathway [38]. In the present study, we report that mitochondrial function was improved in patients supplemented with vitamin D and those with normal levels of vitamin D as compared with patients deficient in it. The activity of CS was 32% lower in the DEF group than in the SUF group and 38% lower compared to the SUPL group. Our data are consistent with the studies undertaken in symptomatic, vitamin D-deficient individuals, which showed that vitamin D therapy augmented muscle mitochondrial maximal oxidative phosphorylation after exercise [39] and increased skeletal muscle CS activity and exercise-mediated cardiorespiratory fitness [40]. Also, recent studies of Ryan and coworkers demonstrated an increased oxygen consumption rate of skeletal muscle cells after treatment with vitamin D, indicating vitamin D action in the regulation of mitochondrial oxygen consumption and dynamics [41]. In addition, our study shows that CS activity was the highest in the SUPL group among men and women as well, when compared with other groups of the same sex. Both women and men in the DEF group had the lowest CS activity among other groups within the same sex. We also found lower protein content of PGC-1*α*, a transcriptional coactivator involved in the formation of slow-twitch fibers and mitochondria biogenesis [42]. This suggests that vitamin D induces PGC-1*α* synthesis and thus may be involved in mitigating muscle atrophy through enhanced mitochondrial function. Although it is well established that the decrease in protein synthesis contributes to disuse atrophy, to date, there has been no data suggesting that PGC-1*α* signalling directly mediates protein synthesis pathways [43]. However, PGC-1*α* transcriptional activity was shown to prevent muscle protein degradation. This was firstly demonstrated by Sandri and coworkers, who showed that overexpression of PGC-1*α* in mice prevented denervation-induced muscle atrophy by preventing the expression of key genes in the ubiquitin proteasome pathway and autophagy [44]. Also, a study on human skeletal muscle showed that PGC-1*α* mRNA is significantly downregulated during both the early and late phases of immobilization-induced muscle atrophy [45]. What is more, the expression of PGC-1*α* in skeletal muscle protects from age-related and denervation-induced muscle atrophy, as well as delays the onset of mitochondrial myopathies [46]. Recently, we demonstrated that vitamin D deficiency caused oxidative stress and higher activity of antioxidant enzymes: manganese superoxide dismutase (MnSOD) and glutathione peroxidase (GPx) in the muscle [14]. Taken together, the lower activity of CS and decreased PGC-1*α* protein content and a higher activity of MnSOD in the muscle indicate impaired mitochondrial function in vitamin D-deficient LBP patients.

As mentioned above, vitamin D acts through the VDR. VDR gene expression is known to be regulated by a variety of hormones including parathyroid hormone, retinoic acid [47], and glucocorticoids [48]. Also, a recent study on HuLM cells showed that estrogen inhibits VDR and that vitamin D has the potential to suppress the expression of estrogen receptor-*α* [49]. Another study reported that 16 weeks of vitamin D intervention induced a 20% increase in human skeletal muscle VDR gene expression in older, mobility-limited, vitamin D-insufficient women [50]. Previously, we presented that VDR muscle content was higher in patients sufficient and supplemented with vitamin D. Furthermore, we showed that lower content of VDR in patients with vitamin D deficiency evokes ROS generation with higher markers of lipid and protein peroxidation as well as increased muscle antioxidant enzyme activity [14]. In order to investigate the possible link between vitamin D and muscle atrophy, we examined PGC-1*α*, FOXO3a, and Akt muscle protein content. FOXO proteins are an important factor in muscle atrophy, which induce the expression of proteasomal genes, MuRF1 and atrogin-1. It is important to note that elevated PGC-1*α* content, besides its function in mitochondrial biogenesis, prevents transcriptional activity of FOXO3a [44], and therefore, the mitochondria might be involved in the progression of skeletal muscle atrophy.

**Figure 7:**
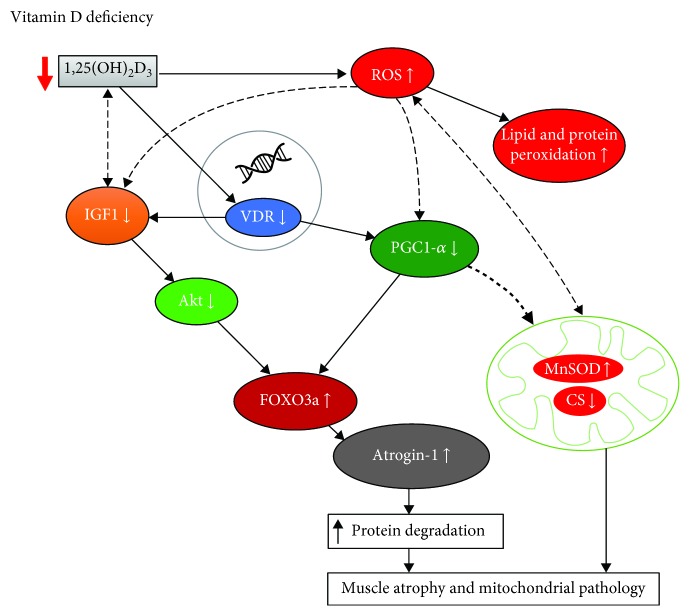
The vitamin D action in the skeletal muscle under the vitamin D deficiency conditions. Bold lines represent established pathways confirmed with our results while thin lines represent possible interactions as a result of vitamin D deficiency. Vitamin D deficiency decreases IGF-1 and PGC-1*α* via VDR—the nuclear receptor. IGF-1/Akt/FOXO3a signalling cascade triggers the muscle atrophy through atrogin-1. ROS generation causes the inhibition of PGC-1*α* and potentially activates FOXO3a thus inducing the muscle atrophy through atrogin-1. The lower protein content of PGC-1*α* directly aggravates mitochondrial biogenesis and function and may cause the oxidative stress. Furthermore, mitochondria are both the source and target of ROS generation. We assume that vitamin D deficiency induces oxidative stress, which is involved and played an important role in muscle atrophy and leads to mitochondrial dysfunction.

Akt blocks the function of FOXO3 by phosphorylation of conserved residues, leading to their sequestration in the cytoplasm away from target genes [51]. Phosphorylated FOXO3a does not translocate to the nucleus, and consequently, the expression of atrogin-1 and MuRF, both target genes of FOXO, is inhibited. It was shown that FOXO3 might also be regulated by the action of peroxisome proliferator-activated receptor gamma coactivator 1-alpha (PGC-1*α*) [44], which is widely accepted to be the master controller of mitochondrial biogenesis as well as a regulator of many genes involved in energy metabolism [52]. In the present study, we found increased levels of Akt and decreased protein content of FOXO3a in the group supplemented with vitamin D as compared to the DEF and SUF groups. Moreover, we observed higher protein content of PGC-1*α* in the muscle of LBP patients with normal vitamin D level and after supplementation with vitamin D. This observation suggests that both vitamin D sufficiency and vitamin D supplementation may contribute to the reversion of atrophic changes. The possible mechanism of action of vitamin D in the skeletal muscle still needs to be addressed in future studies. According to one proposed model, in cases of muscle atrophy associated with disuse, decreases in IGF-1 cause the inhibition of Akt and dephosphorylation of FOXO3a. Dephosphorylated FOXO3a translocates to the nucleus and promotes the expression of atrogin-1 and MuRF1 and subsequently accelerates the degradation of muscle proteins [10]. Our data confirms that the possible action of vitamin D in the prevention of muscle atrophy may be mediated through the IGF-1/Akt/FOXO3a pathway. Nevertheless, with no changes in serum IGF-1 in the supplemented patients, our observations indicate that PGC-1*α* and mitochondria may play a crucial role in muscle atrophy through regulating mitochondrial function. Also, as mentioned above, PGC-1*α* might inactivate FOXO3a and therefore contribute to resisting muscle atrophy and restore the physiological functions of the mitochondria. Moreover, it was shown that FOXO3a activation caused the upregulation of MnSOD gene expression and downregulation of mitochondrial gene expression [53]. Our previously published data confirms this observation. We reported increased MnSOD activity in vitamin D-deficient patients. Taken together, our findings show that the action of vitamin D may be mediated through the IGF-1/Akt/FOXO3 pathway or through PGC-1*α* and FOXO3a independently (Figure 7).

In summary, we show that vitamin D deficiency is associated with attenuated CS activity, decreased protein content of PGC-1*α*, and previously published oxidative stress in the multifidus muscle of LBP patients [14]. We detected increased protein content of atrogin-1, in the muscle of women with lower vitamin D level. These results suggest that vitamin D deficiency induces muscle atrophy and reduces mitochondrial function in the paraspinal muscle. In addition, we observed higher IGF-1 content in both serum and muscle in patients with sufficient vitamin D level. Our results suggest that the action of vitamin D in the muscle may be triggered through either the Akt/FOXO3a pathway or PGC-1*α* and mitochondria. Supplementation with vitamin D to sufficient serum vitamin D level in LBP patients increased mitochondrial function and inhibited muscle atrophy in the multifidus muscle, and it may have a beneficial impact on an effective early rehabilitation in LBP patients. However, future studies on muscular function should also consider the supplementation of patients sufficient in vitamin D and patients with different BMI and age for better understanding of the mechanism of vitamin D function. There should be patients' stratification according to BMI and different hormonal and physiological gender responses.

## Figures and Tables

**Table 1:** Characteristics of LBP patients.

	Age	BMI	25(OH)D_3_ (nmol/L) Before	25(OH)D_3_ (nmol/L) After	*p*
DEF (*n* = 14)	49.7 ± 2.6	30.3 ± 0.9	39.8 ± 2.4	38.2 ± 2.1	n.s
F (*n* = 6)	51.2 ± 5.2	28.0 ± 0.8	37.6 ± 4.2	36.9 ± 3.9	n.s
M (*n* = 8)	48.8 ± 2.5	32.3 ± 1.1	41.5 ± 3.0	39.1 ± 2.4	n.s
SUF (*n* = 10)	45.8 ± 3.1	27.9 ± 0.9	73.3 ± 2.9^∗^	72.5 ± 6.8^#^	n.s
F (*n* = 5)	45.8 ± 2.6	27.3 ± 1.4	71.5 ± 5.2^∗^	72.1 ± 7.1^#^	n.s
M (*n* = 5)	45.8 ± 6.0	28.5 ± 1.2	75.1 ± 3.1^∗^	72.9 ± 12.5^#^	n.s
SUPL (*n* = 14)	48.2 ± 2.8	28.5 ± 1.4	52.8 ± 3.0	86.6 ± 3.2	<0.005
F (*n* = 8)	50.5 ± 3.4	28.1 ± 1.9	50.8 ± 3.8	85.1 ± 4.0	<0.005
M (*n* = 6)	45.2 ± 4.9	29.4 ± 0.4	55.4 ± 4.9	88.7 ± 5.3	<0.005

Values are the means (±SEM). F: female; M: male. ^∗^ *p* < 0.001—difference between the indicated result/mean and DEF and SUPL groups at the same time point. ^#^ *p* < 0.001—difference between the indicated result/mean and DEF group at the same time point.

## Data Availability

The data used to support the findings of this study are included within the supplementary information file.

## Abbreviations

Akt: Serine/threonine-specific protein kinase
Cu/ZnSOD: Copper/zinc-dependent dismutase
CS: Citrate synthase
FOXO3: Forkhead box O3
GPx: Glutathione peroxidase
IGF-1: Insulin-like growth factor 1
LBP: Low back pain
MnSOD: Manganese-dependent superoxide dismutase
PGC-1*α*: Peroxisome proliferator-activated receptor gamma coactivator 1-alpha
PLIF: Posterior lumbar interbody fusion
ROS: Reactive oxygen species
VDR: Vitamin D receptor.

## Supplementary Materials

Supplementary Materials GR: groups; DEF: deficient; SUF: sufficient; SUPL: an adequate level of vitamin D in serum; Serum IGF-1 before: serum insulin-like growth factor before supplementation with vitamin D; Serum IGF-1 after: serum insulin-like growth factor after supplementation with vitamin D; IGF-1: the muscle insulin-like growth factor after supplementation with vitamin D and placebo; CS: citrate synthase activity in the muscle after supplementation with vitamin D and placebo; atrogin-1; the muscular concentration of atrogin-1, measured with ELISA; Akt: the ratio of phosphorylated and dephosphorylated serine/threonine-specific protein kinase versus loading control, *β*-tubulin, measured in the muscle after supplementation with vitamin D and placebo; FOXO3: the ratio of phosphorylated and dephosphorylated forkhead box O3 versus loading control, *β*-tubulin, measured in the muscle after supplementation with vitamin D and placebo; PGC-1*α*: the muscular peroxisome proliferator-activated receptor gamma coactivator 1-alpha versus loading control, *β*-tubulin, measured in the muscle after supplementation with vitamin D and placebo; Fbx32 (atrogin-1): the muscular protein content of atrogin-1 versus loading control, *β*-tubulin, measured in the muscle after supplementation with vitamin D and placebo.
