# Supplementary material for: Vitamin D Deficiency Is Associated with Muscle Atrophy and Reduced Mitochondrial Function in Patients with Chronic Low Back Pain
Source: Oxid Med Cell Longev. 2019 Jun 2;2019:6835341. doi: 10.1155/2019/6835341 (PMC6589343; doi:10.1155/2019/6835341)
Supplement: Supplementary Materials — GR: groups; DEF: deficient; SUF: sufficient; SUPL: an adequate level of vitamin D in serum; Serum IGF-1 before: serum insulin-like growth factor before supplementation with vitamin D; Serum IGF-1 after: serum insulin-like growth factor after supplementation with vitamin D; IGF-1: the muscle insulin-like growth factor after supplementation with vitamin D and placebo; CS: citrate synthase activity in the muscle after supplementation with vitamin D and placebo; atrogin-1; the muscular concentration of atrogin-1, measured with ELISA; Akt: the ratio of phosphorylated and dephosphorylated serine/threonine-specific protein kinase versus loading control, β-tubulin, measured in the muscle after supplementation with vitamin D and placebo; FOXO3: the ratio of phosphorylated and dephosphorylated forkhead box O3 versus loading control, β-tubulin, measured in the muscle after supplementation with vitamin D and placebo; PGC-1α: the muscular peroxisome proliferator-activated receptor gamma coactivator 1-alpha versus loading control, β-tubulin, measured in the muscle after supplementation with vitamin D and placebo; Fbx32 (atrogin-1): the muscular protein content of atrogin-1 versus loading control, β-tubulin, measured in the muscle after supplementation with vitamin D and placebo. [file 6835341.f1.pdf]

GR	Sex	Age	BMI	serum IGF-1 [ng/ml] Before	serum IGF-1 [ng/ml] After	muscle IGF-1 [ng/ml]	CS [nmol/min/mg protein]	Atrogin-1 [ng/mg protein]	pAkt/Akt [a.u]	FOXO3a/pFOXO3a [a.u]	PGC-1a [a.u]	Fbx32 (Atrogin-1) [a.u]
DEF	F	52	27,2	137,78	117,92	52,19	65,30	86,61				
DEF	F	64	31,3	66,31	62,34	25,27	49,90	36,72				
DEF	F	53	27,3	81,62	158,76	48,83	32,00	100,67				
DEF	F	33	27,0	145,15	105,45		35,10	33,30	0,78	1,18	0,00	0,22
DEF	F	65	29,0	116,22	77,08		7,70	24,23	1,24	39,01		
DEF	F	40	26,2	115,09	102,61		59,00	50,69			0,29	0,08
DEF	M	50	32,3	107,71	111,12	77,14	59,40	17,07				0,07
DEF	M	57	31,8	108,85	92,97	16,75	37,70	18,89	1,35	6,84		
DEF	M	56	31,4	75,95	130,40	16,98	30,30					
DEF	M	42	32,0	104,31	72,55							0,10
DEF	M	41	33,1				37,70	8,61	1,20	3,24		0,12
DEF	M	56	30,4	95,80	111,68		54,50	18,63	0,83	0,99	0,23	0,14
DEF	M	40	28,8	111,65	106,38		25,40	19,97				
DEF	M	48	38,3	86,73	75,95		47,00	12,44	1,00	0,30	0,05	0,07
SUF	F	52	32,3	183,15	215,48	61,96	64,40	20,64	1,31	2,51	0,42	0,12
SUF	F	46	23,8	116,79	108,85	53,91	88,10	24,24				
SUF	F	51	27,9	115,66	110,55	87,76	101,60	31,10				
SUF	F	39	26,4	123,28	127,37		14,50	10,16	1,13	0,28	1,00	0,02
SUF	F	41	26,3	130,40	108,28		4,10	27,24				
SUF	M	34	27,4	146,85	89,56	84,13	101,40	37,69				0,02
SUF	M	65	24,6	108,28	95,80		43,00	23,48				
SUF	M	34	30,2	134,37	154,79		44,30	24,91	0,89	3,19	0,28	0,14
SUF	M	54	28,7				91,90	11,95	0,47	1,54	0,52	0,13
SUF	M	42	31,7	134,37	157,06			19,18			0,31	0,13
SUPL	F	63	30,5	109,33	98,88	13,65	54,30	30,64				
SUPL	F	50	23,6	110,55	102,61	72,32	89,60	45,90	1,23	0,71		
SUPL	F	43	24,8	114,52	143,45	15,33	40,30	50,56				
SUPL	F	57	20,1	137,21	102,61	59,50	64,70	37,36				
SUPL	F	41	32,1	83,32	78,79	43,97	33,30	10,99				
SUPL	F	61	28,1	82,76	78,79	43,58	110,60	46,35				
SUPL	F	52	37,7	86,16	60,07		35,70	23,43			0,15	
SUPL	F	37	27,9	102,61	83,89			10,91	1,56	0,28	0,41	0,02
SUPL	M	44	29,1	130,40	123,03		110,80	12,17				
SUPL	M	35	27,8	112,25	112,25	28,73	54,70	29,17	1,46	0,61	0,02	0,10
SUPL	M	33	30,7	109,00	94,10	57,27	86,80	20,85	1,68	0,45	0,65	0,08
SUPL	M	41	29,9	110,55	115,66		44,40	13,87			0,39	0,08
SUPL	M	53	29,6	113,95	102,04		73,10	6,47	1,20	0,18	0,66	0,09
SUPL	M	65	29,2	113,39	146,85		82,20	8,68	1,39	0,87		0,11
